# Integrated iPRISM Direct-on-Urine Platform for Rapid UTI Diagnosis in a Double-Blind Clinical Trial

**DOI:** 10.1021/acsmeasuresciau.5c00187

**Published:** 2026-02-18

**Authors:** Xin Jiang, Ramy Fishler, Gali Ron, Keren Boguslavsky, Sarel Halachmi, Ester Segal

**Affiliations:** † Department of Biotechnology and Food Engineering, 26747 Technion − Israel Institute of Technology, Haifa 3200003, Israel; ‡ Department of Urology, Bnai Zion Medical Center, Haifa 3104800, Israel; § The Faculty of Medicine, Technion − Israel Institute of Technology, Haifa 3525433, Israel

**Keywords:** optical sensor, bacteriuria screening, infection screening, antimicrobial susceptibility testing, diffraction grating, urinary tract infection

## Abstract

Rapid point-of-care (POC) diagnostics for urinary tract infections (UTIs) are critical for targeted therapy and antibiotic stewardship. We report the first double-blind study of a POC diagnostic system for UTI detection and phenotypic antimicrobial susceptibility testing (AST), using the label-free, real-time iPRISM platform (intensity-based phase-shift reflectometric interference spectroscopic measurement), which traps and grows bacteria on photonic silicon chips. In this near-patient study, unprocessed urine samples were tested in a single-use microfluidic device that integrates both infection screening and AST. Infection screening achieved 97% sensitivity and 60% specificity within 90 min; threshold optimization at 75 min improved performance to 81% specificity and 82% sensitivity. For AST, iPRISM correctly classified 100% of gentamicin-exposed samples in just 30 min and achieved 62% sensitivity and 87% specificity for ciprofloxacin within 90 min. Notably, our preliminary data also demonstrate the potential to differentiate between fungal and bacterial infections, thereby broadening its diagnostic applicability. iPRISM delivers clinically actionable results within a relevant time frame, enabling single-visit prescriptions and supporting personalized, data-driven UTI management.

## Graphical abstract

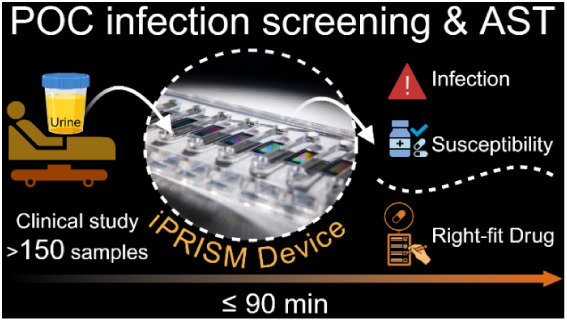

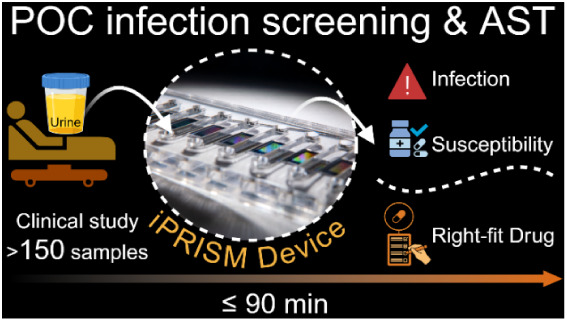

## 1 Introduction

Urinary tract infections (UTIs) pose a significant global health and economic burden with over 400 million cases and more than 200,000 associated deaths worldwide annually.^1–4^ The growing prevalence of antimicrobial resistance (AMR) underscores the urgent need for rapid and accurate diagnostic solutions that support clinical decision-making and facilitate patient-tailored antimicrobial therapy.^5–7^ Recognizing this, the World Health Organization (WHO) has identified improved diagnostics as a key research priority in its Global Research Agenda for Antimicrobial Resistance in Human Health.^8^

An example of this clinical need is evident in the diagnosis of UTIs, which remain among the most common bacterial infections globally.^9,10^ To date, gold standard methods for UTI diagnosis continue to rely heavily on culture-based techniques,^6,10–12^ which are slow and labor-intensive, often requiring several days to deliver results. Diagnostic accuracy is often limited as detection thresholds may vary by clinical context and the frequent need for expert interpretation.^13,14^ Furthermore, standard urine culture may often miss fastidious or less common uropathogens such as *Streptococcus agalactiae* (*S. agalactiae*), and *Candida* species.^15,16^ Together, these limitations delay definitive diagnosis and complicate early clinical decision-making.

A critical subsequent component of the clinical workflow is antimicrobial susceptibility testing (AST), which guides the selection of effective antibiotic therapy and supports antimicrobial stewardship.^17–19^ For this step, a single colony from the overnight culture plate is transferred to a sterile growth medium and adjusted to a standardized inoculum density, followed by AST which determines the minimum inhibitory concentration (MIC) of an antimicrobial agent. This step requires additional 8–24 h, depending on the technique used, further delaying therapeutic decision-making.^20–22^ A recent Microcolony-seq study revealed that host-acquired phenotypic memory in human pathogens including *Escherichia coli*­(*E. coli*) and *Staphylococcus aureus* (*S. aureus*) is erased when bacteria reach a stationary phase, further complicating diagnosis. Thus, raising concern as for the reliability of conventional AST protocols, since overnight enrichment may eliminate host-adapted phenotypic states present during active infection.^23^ Compounding these limitations, inappropriate antibiotic prescribing remains a major concern in UTI management.^24^ According to a recent report,^25^ 77% of antibiotic prescriptions for UTIs were inappropriate and did not align with recommended clinical practices, such as incorrect drug selection. This, in combination with the delays in diagnosis, underscores the urgent need for rapid and accessible diagnostic tools that are culture-free and direct-from-sample.^26–28^

Profound progress has been achieved in developing direct-from-urine diagnostic technologies, targeting bacteriuria screening, pathogen identification, and AST, with more than 30 diagnostic platforms currently in various stages of research or commercialization.^5,29,30^ However, most of these technologies only partially addressed the clinical workflow, typically at detection or identification and with limited integration of AST. Clinical translation remains limited as many studies were validated on spiked urine samples,^29,31–33^ imposed strict exclusion criteria,^34,35^ or required additional preprocessing steps such as centrifugation and filtration.^26,34–36^ Based on the current literature, only a limited number of reported systems have been evaluated using blinded urine samples,^28,37^ a critical step toward clinical translation. Consequently, analytical performance demonstrated under controlled conditions often overestimate performance in real-world clinical settings.

Despite the significant advances in sensor and biosensor technologies for bacterial detection and AST in general,^21,38–42^ and for UTIs in particular,^10,12,43–45^ fully integrated platforms capable of performing both functions remain limited.^28,46,47^ For example, Liao et al. demonstrated 100% sensitivity for Gram-negative uropathogen detection via an electrochemical sandwich assay, but only following centrifugation and bacterial lysis.^48^ 15 years later, their system evolved into a phenomolecular UTI platform that combines pathogen detection and AST within 30 min.^46,49^ Yet, this labeled system still required filtration, involved costly and fragile molecular probes, and has not yet been validated in a double-blind setting. Zhang et al. introduced an innovative large volume solution scattering imaging approach combined with image analysis, achieving impressive results, 100% specificity and 93% sensitivity in screening along with 100% agreement for ciprofloxacin AST within 60 min, yet still requiring extensive sample preparation.^28^ Collectively, these studies underscore the promise of integrated rapid infection screening and AST for UTI diagnostics.^28,33,46,49^ Nevertheless, the need for extensive preprocessing, labeling, and double-blind evaluation remains a major obstacle to clinical implementation. Accordingly, a platform that functions directly on unprocessed clinical urine and demonstrates reliable performance in a rigorous, double-blind, on-site study would be of considerable value.

Building on our previous proof-of-concept work, where we introduced the intensity-based phase shift reflectometric interference spectroscopic measurements (iPRISM) method for AST and demonstrated its performance with confirmed*E. coli* infected urine,^50^ we now report a streamlined, integrated platform that performs both infection screening and AST simultaneously on fresh urine samples suspected of infection. iPRISM is a real-time optical sensing technique that detects microbial infections by monitoring microbial growth dynamics on microstructured silicon photonic chips.^50–52^ This method relies on patterned silicon gratings that both facilitate microbial capture and function as integrated optical transducers.^50,51,53^ As microbes colonize and proliferate within the silicon microstructures in the presence of varying antimicrobial concentrations, their presence modulates the reflectance interference signal, enabling real-time, label-free quantification of growth under antimicrobial exposure and direct classification of susceptibility phenotypes.^50,51,54^

In this study, iPRISM is applied directly to freshly collected, unprocessed human urine, thereby eliminating preprocessing steps, reducing turnaround time, and integrating UTI screening and AST into a single assay. By addressing key limitations of existing diagnostic approaches, including reliance on culture, extensive sample preparation, and fragmented workflows, this platform supports integration into routine clinical diagnostic workflows. Most importantly, we conducted a double-blind, on-site, near-patient clinical trial comparing our rapid UTI diagnosis and AST technique with standard diagnostic procedures. This study design enables an objective evaluation of performance under clinically relevant conditions, an essential benchmark for translation. This methodology adheres to established antibiotic breakpoint concentrations, aiming to provide rapid, accurate, and clinically actionable UTI diagnosis at the POC.

## 2 Experimental Section

### 2.1 Materials

(3-Aminopropyl)­triethoxysilane (APTES), gentamicin sulfate, and ciprofloxacin, were purchased from Sigma-Aldrich, Israel. Absolute ethanol was supplied by Merck, Germany. Acetone was supplied by Gadot, Israel. Acetic acid and isopropyl alcohol were supplied by Bio-Lab Ltd., Israel. Photoresist AZ4533 was supplied by Metal Chem Ltd., Israel. Brain heart infusion (BHI, 237500) broth dehydrated medium was obtained from Difco, USA.

### 2.2 Preparation of Solutions and Media

BHI medium (37 g L^–1^) was prepared according to manufacturer’s instructions in Milli-Q water (18.2 MΩ·cm) and was autoclaved at 121 °C for 15 min prior to use.

*Clinical Samples*: Anonymous urine samples were collected from patients hospitalized at Bnai Zion Medical Center (Haifa, Israel, ethnics approval: BNZ 0110-14). From May 2022 to September 2023, specimens were obtained from the clinical microbiology laboratory; between September 2023 and January 2025, they were collected from the Urology Department. Most specimens were tested immediately after standard microbiological cultures; when immediate testing was not feasible, samples were refrigerated at 4 °C for a maximum of 24 h prior to iPRISM analysis. All clinical tests were conducted by certified technicians at the clinical microbiology laboratory, with infections diagnosed using gold-standard urine culture methods. Pathogen identification was performed via traditional culturing techniques or matrix-assisted laser desorption/ionization time-of-flight mass spectrometry (MALDI-TOF MS; Bruker, Germany). Antimicrobial susceptibility testing was conducted using the VITEK 2 automated system (bioMérieux, France). All clinical results were reported by Bnai Zion Medical Center within a minimum of 3 days.

### 2.3 Fabrication of iPRISM Devices

The silicon photonic chips featured a periodic porous microstructure, consisting of an array of square wells measuring 3 μm in width, 1 μm in spacing, and 4 μm in depth. Fabrication was performed on 4-in. silicon wafers (Siltronix, France) coated with a ∼1000 Å thermally grown SiO_2_ hard mask. Prior to photolithography, wafers were treated with vaporized hexamethyldisilazane (HMDS) as an adhesion promoter, followed by spin-coating of the positive photoresist AZ1512 using an automatic coater (Delta 80 RC, SUSS MicroTec, Germany) and soft baking at 110 °C for 90 s. The desired microstructure pattern was defined by laser lithography using the Maskless Aligner MLA 150 (Heidelberg Instruments, Germany). After exposure, the photoresist was developed using 10% tetramethylammonium hydroxide (TMAH) in an automated developer (Delta 8+, SUSS MicroTec, Germany). The SiO_2_ hard mask was opened at the patterned locations by reactive ion etching (RIE) with CHF_3_/O_2_ on a Plasma-Therm Etching System 790 (Plasma-Therm LLC, USA). Subsequently, deep reactive ion etching (DRIE) using SF_6_ and C_4_F_8_ was performed on a Plasma Etcher Versaline (Plasma-Therm LLC, USA) to define the microwell depth. Residual photoresist and hard mask materials were removed using sequential treatments with 1-methyl-2-pyrrolidone (NMP), MLO 07, piranha solution (H_2_SO_4_:H_2_O_2_ = 2:1), and buffered oxide etchant (BOE), followed by RCA cleaning, including diluted HF and NH_4_OH/H_2_O_2_/H_2_O treatments. The wafer was then thermally oxidized at 800 °C for 1 h in a furnace (BTI-Bruce RTRI-878) to form a ∼70 Å thermally grown SiO_2_ layer. All fabrication processes were carried out at the Micro-Nano Fabrication and Printing Unit, Technion. To protect the delicate microstructures during dicing, the samples were precoated with photoresist before being diced using an automated dicing saw, DAD3350 (Disco, Japan), yielding 4 × 4 mm or 6 × 58 mm silicon chips. These chips were integrated into 7.6 × 2.5 cm injection molded poly­(methyl methacrylate) microfluidic devices containing ten microchannels, each with a capacity of 60 μL and a channel height of 1.2 mm constructed by Potomac Photonics, Inc. (Maryland, USA). The silicon chips were integrated within the device using pressure-sensitive adhesive and subsequently functionalized by exposure to a 2% (v/v) solution of APTES prepared in 50% ethanol for 1 h at room temperature, followed by thorough rinsing with 70% ethanol and drying with nitrogen. The devices were then stored in a desiccator for up to 2 weeks prior to use.

### 2.4 iPRISM Assay

Clinical urine samples were mixed with BHI, with or without an antimicrobial agent, at a 1:1 volume ratio without preincubation. The final antimicrobial concentrations were 8 μg mL^–1^ for gentamicin and 0.06 μg mL^–1^ for ciprofloxacin, which were found to be the iPRISM breakpoint concentrations for determining antibiotic resistance of *E. coli*.^50^ The resulting suspensions were introduced into each channel of the iPRISM device (see Figure S1a) after disinfecting it with 70% ethanol and both inlet and outlet ports were sealed using a Breath-Easy membrane (Z380059; Sigma-Aldrich). The device was fixed to a heat-controlled aluminum substructure maintained at 37 °C via a 40 °C water bath to compensate for heat loss, connected to a motorized linear stage (MTS50-Z8, Thorlabs, Inc., USA) for single-axis movement control. For optical measurements (Figure S1a), a 74-UV collimating lens connected to a bifurcated fiber optic cable (Ocean Optics, USA) was positioned perpendicular to the device, illuminating the photonic silicon microstructure via an HL-2000 white light source (Ocean Optics, USA) and the reflected light was recorded by a USB4000 CCD spectrometer (Ocean Optics, USA). Reflectance spectra, exhibiting interference fringes (Figure S1b) due to reflections at the top and bottom interfaces of the porous silicon structure (Figure S1c), were continuously collected using LabView (National Instruments, USA) over a minimum duration of 90 min. Each spectrum was obtained by averaging 375 consecutive scans, with an integration time of 20 ms per scan. Acquired reflectance spectra in the range of 450–900 nm were analyzed using Fast Fourier Transform (FFT) in MATLAB software (R2024a), after resampling onto a uniformly spaced inverse-wavelength axis and applying a Hanning window with zero padding to enhance Fourier-space resolution. The FFT-shifted transform was then used to extract a single dominant reflection peak for each measurement (Figure S1d). The peak amplitude serves as the primary signal, providing a rapid optical readout of microbial activity at the biosolid interface.^50,55^ The decrease in peak amplitude (denoted as *–*Δ*I*) over time was calculated using the following equation:

$$-\Delta I\left(\%\right)=-\frac{I-I_{15}}{I_{15}}\times 100\%$$ 1

in which *I*_15_ represents the peak amplitude of the Fourier transformed spectrum after an initial 15 min incubation of the chip with the respective samples studied in the iPRISM device. This short conditioning time allows microbes to settle within the Si microstructures as demonstrated in our previous study.^50,51^

### 2.5 Electron Microscopy

For high-resolution scanning electron microscopy (HR-SEM) imaging, iPRISM devices were disassembled via immersion in 70% ethanol for 2 days, then silicon chips were autoclaved in accordance with safety regulations. Zeiss Ultra Plus high-resolution scanning electron microscope equipped with a Schottky field-emission gun (Carl Zeiss, Germany) at an acceleration voltage of 1 kV was employed. In selected micrographs, bacterial cells were false-colored using Adobe Photoshop CS3 for clarity.

### 2.6 Statistical Analysis

Sensitivity, specificity and overall accuracy against the clinical reference methods were calculated to evaluate the iPRISM assay performance. For AST, very major errors (VME) and major errors (ME) were calculated which stand for the percentage of false susceptible and false resistant results compared to VITEK 2, respectively. Receiver operating characteristic (ROC) curve analyses were performed by plotting sensitivity against (1 – specificity) across multiple diagnostic thresholds, with the area under the curve (AUC) to assess the overall iPRISM diagnostic accuracy. The Youden index (sensitivity + specificity – 1) was calculated to identify optimal diagnostic threshold. OriginPro 2025 (student version) was used for calculating the p-value and 95% confidence interval for the AUC in comparison to random classification.^56^

## 3 Results and Discussion

### 3.1 Integrated iPRISM Platform and Study Design

In this study, we advance the iPRISM system to perform simultaneous pathogen detection and AST directly from unprocessed urine samples. Figure 1a outlines the concept of the integrated iPRISM assay, in which microstructured silicon photonic chips transduce microbial colonization and growth dynamics into real-time optical interference signals, thereby supporting both infection screening and susceptibility profiling. To showcase its diagnostic performance for UTIs, the iPRISM system was deployed on-site at the Urology Department of Bnai Zion Medical Center in a prospective, double-blind study. This near-patient bedside testing directly at the POC minimizes preanalytical variability associated with urine transport. In parallel, enrolled samples were also independently analyzed by the hospital’s microbiology laboratory using the standard multistep workflow of urine culture, pathogen identification, and AST, which typically requires 3 to 7 days to complete (Figure 1b).

**Figure 1:**
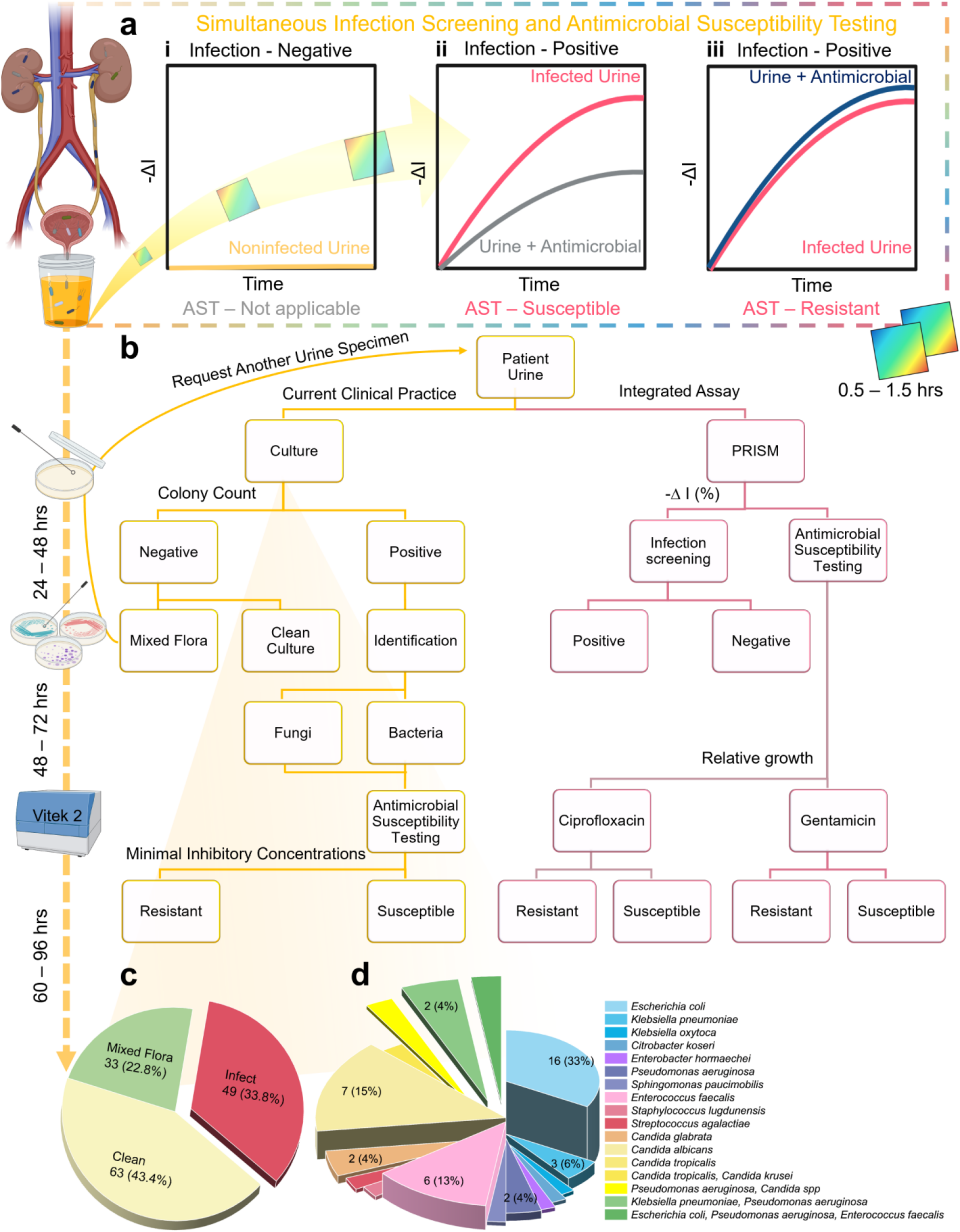
Schematic overview of iPRISM for rapid UTI diagnosis and comparative workflow analysis. (a) iPRISM enables simultaneous infection screening and AST directly on clinical urine specimens. Three infection scenarios can be distinguished based on the measured signal: (i) negative infection, (ii) positive infection with an antimicrobial-susceptible uropathogen, and (iii) positive infection with an antimicrobial-resistant uropathogen. (b) Overall flow of iPRISM and clinical routine UTI diagnostics. Current clinical UTI workflow requires sequential steps: (1) standard urine culture (24–48 h for screening), (2) subculture for microbial identification, and (3) AST (e.g., using VITEK 2), with turnaround time from sample to results ranging from 3 to 7 days. The iPRISM system described herein proceeds without culturing, achieving diagnosis within 90 min. (c) Distribution of infection scenario of the collected clinical urine specimens. (d) Isolated causative agents of culture-positive specimens (derived from the red slice in c), categorized by Gram-negative bacteria (blue-purple), Gram-positive bacteria (pink-red), fungal species (yellow-orange), and polymicrobial infections (yellow-green). Created with BioRender.

The core of the iPRISM assay is a micropatterned silicon chip that is chemically functionalized to promote nonspecific capture of microorganisms from the test suspension, enabling colonization and proliferation of microbial species on the silicon surface. Briefly, the silicon chips are designed to accommodate a broad range of bacterial species, featuring a periodic array of square-shaped pores of approximately 3 μm wide, 4 μm deep, and separated by ∼1 μm-thick walls. The silicon chip surface is amine-functionalized, as our previous studies have shown that this modification enhances microbial adhesion under these conditions.^50,55^ To initiate the assay, urine specimens were mixed at a 1:1 volume ratio with brain heart infusion (BHI) broth with or without antibiotics and then loaded into the iPRISM microfluidic channels. Final antibiotic concentrations corresponded to pre-established breakpoint concentrations established for *E. coli*.^51^ By that, we omitted the previous prewarming step for those retrieved samples (stored at 4 °C for ≥48 h) and the optical density (OD_600_) standardization, further minimizing sample processing.^50^ During the assay, the microfluidic device was maintained at 37 °C and the raw spectra were continuously collected. Fourier transform analysis was then used to extract useful information, which was eventually used to calculate the −Δ*I* (%) values that correlate with bacterial growth.

To correlate microbial activity on the micropatterned photonic silicon chip with a positive detection signal, time-resolved optical changes are expressed as −Δ*I* (%), reflecting the decrease in light intensity over time due to microbial presence. In this integrated assay, a constant optical signal that remains unchanged throughout the observation period indicates the absence of uropathogens (Figure 1a-i), whereas the presence of viable pathogens is detected by an increase in −Δ*I* (%) values, reflecting microbial colonization and proliferation on the patterned silicon surfaces (pink traceline in Figure 1a-ii, (iii). When susceptible pathogens encounter effective antimicrobials, growth suppression results in a smaller increase in *–*Δ*I* (%) values (Figure 1a-ii). Conversely, in cases of antimicrobial resistance, microbial proliferation persists, leading to unchanged optical signal intensity (blue traceline in Figure 1a-iii). For further details on the assay, see Section 2.4 and our previous work.^50,51,53,55,57^

### 3.2 Overview of Clinical Urine Specimens and Distribution of Pathogens Detected by the Traditional Culture Approach

A total of 154 urine samples were collected from hospitalized patients suspected of UTI at Bnai Zion Medical Center between May 2022 and January 2025. Of these, 145 samples underwent parallel testing with both the reference method and the iPRISM assay, including cases later confirmed as complicated UTIs through clinical evaluation. Note that nine samples were excluded from the analysis due to data inconsistencies caused by technical malfunctions during the experimental runs. These issues are attributed to system deployment at bedside rather than in a controlled lab environment.

The collected samples represent a diverse patient population, with ages ranging from 17 days to 101 years (Table S1). Ninety-one (63%) urine specimens were from male patients and 53 (37%) from female patients (Table S1). The sources of urine samples varied among patients, including midstream voids, nephrostomies, ureteral catheters, and bladder catheters. Owing to these differences and the underlying medical conditions, urine samples exhibited diverse physical properties, where urine transparency ranged from clear to turbid or foamy, while color varied from colorless to dark brown. Hematuria was observed in some cases, causing urine to appear pink to dark brown instead of its normal pale-yellow color.

The clinical human urine handling procedures of the current study are schematically depicted in Figure 1b. After urine collection, each specimen was divided into two portions. One portion underwent the standard clinical workflow for microbial infection screening, pathogen identification and AST, while the other was analyzed using the iPRISM assay. It should be emphasized that these two analyses were carried out simultaneously and independently of each other.

For the iPRISM analysis, the resulting −Δ*I* (%) signal was then used both for infection detection and AST. Since our method does not rely on pathogen identification, as in the standard clinical work flow, urine samples were analyzed using the iPRISM predetermined threshold, established in a previous work for the most common uropathogen*E. coli*.^51^ Importantly, for the urine analyzed using standard clinical procedures, clinicians diagnosed UTI based on the established microbial thresholds (varied for uropathogen type and patient conditions) after at least 24 to 48 h of incubation.^58^ Of the 145 samples analyzed by the microbiology laboratory, 96 (66%) were classified as non-UTI cases, including 63 samples with clean cultures and 33 with mixed microbial growth (Figure 1c). The remaining 49 samples (34%) showed positive uropathogen growth, with 43 monomicrobial and 6 polymicrobial infections (Figure 1c). Figure 1d summarizes the identified causative pathogens, a total of 56 microbial strains were isolated, including 33 Gram-negative bacteria (59%), 10 Gram-positive bacteria (18%), and 13 fungal strains (23%). Gram-negative bacteria were the most prevalent group, where *E. coli* was the most common species (25%). This distribution is consistent with previous microbial diversity analyses from the Urology Department at Bnai Zion Medical Center^21^ and agree with the well-established general observation of *E. coli* as the leading cause of UTIs.^2,4,59^ Moreover, prior studies have shown that community-acquired*E. coli* UTI pathogens are more frequently isolated than hospital-acquired strains.^60,61^ In the present study, fungal infections constituted a larger proportion of UTIs than*Enterococcus faecalis* (*E. faecalis*), the most common Gram-positive uropathogen. This trend aligns with the rising prevalence of fungal UTIs as reported in both European and Chinese studies,^62,63^ and is driven by multiple factors such as the widespread use of broad-spectrum antibiotic, increased use of indwelling catheters, prolonged hospitalization, and a growing population of immunocompromised individuals, including those with diabetes mellitus or receiving immunosuppressive therapy.^64–67^

### 3.3 iPRISM Assay for UTI Screening

In this study, iPRISM was used to monitor microbial growth in real time by tracking changes in reflected light diffraction patterns as manifested by −Δ*I* (%) (see Section 2.4). As depicted in Figure S2a, a gradual increase in −Δ*I* (%) was typically observed for triplicate tests of infected samples, indicating bacteriuria. In contrast, noninfected urine specimens generally did not show this trend (Figure S2b).

Figure 2 depicts characteristic iPRISM plots, demonstrating that across samples, the real-time *–ΔI* (%) response exhibited variability in both the slope and magnitude. This variability reflects differences in microbial load as well as bacterial growth kinetics, ascribed to the design of this double-blind clinical study. This broader range of pathogens and microbial loads challenged the iPRISM assay, as some exhibited rapid exponential growth phases, while others demonstrated delayed onset or reduced growth rates. Despite this biological heterogeneity, iPRISM demonstrated its capability in detecting infections across multiple taxonomic groups. Representative examples are shown for*E. coli*, *Klebsiella spp.*, *E. faecalis*, and *S. agalactiae* (Figure 2a–d, respectively). HR-SEM confirmed that microbes were physically trapped within the micropatterned silicon and that the observed optical response arose from their physical presence (Figure 2e–h).

**Figure 2:**
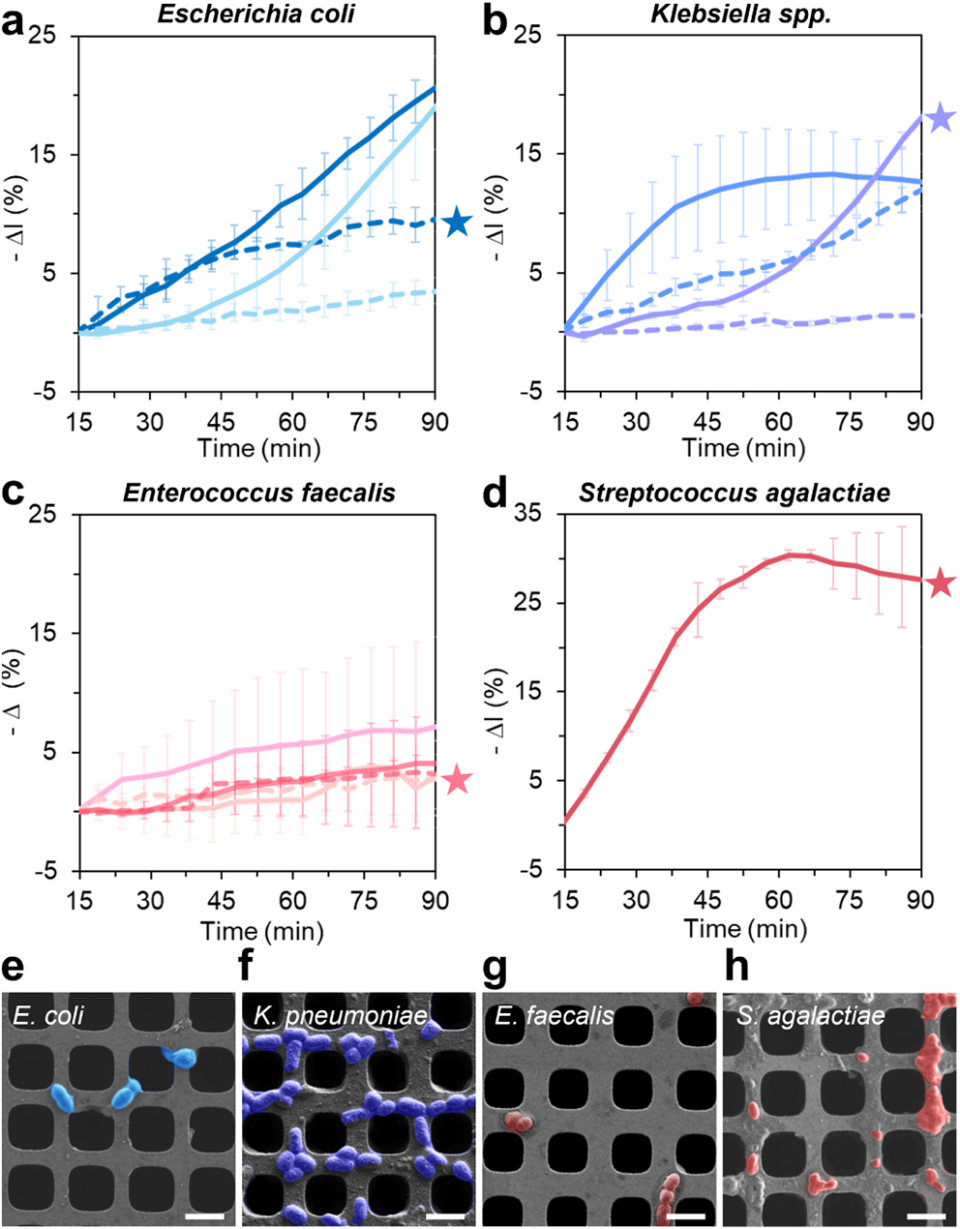
iPRISM for UTI detection directly on clinical human urine samples. (a–d) Representative real-time characteristic bacterial growth curves as measured by the iPRISM assay for (a) *Escherichia coli*, (b) *Klebsiella* spp., (c)*Enterococcus faecalis*, and (d) *Streptococcus agalactiae*. (e–h) Corresponding HR-SEM images of the bacterial species in panels a–d, respectively, demonstrating their spatial distribution on the surface of microwells for retrieved silicon chips from the iPRISM device (for each bacterium, the curve corresponding to the image is marked with a star). Scale bars denote 3 μm.

To evaluate the iPRISM infection screening performance, an initial classification strategy was based on a fixed threshold of −Δ*I* (%) = 0, based on the rationale that a positive growth signal reflects microbial activity.^50^ Among 101 clinical urine samples, iPRISM achieved 97% sensitivity at 90 min (Figure 3a-iii), increasing from 79% at 30 min (Figure 3a-i). This trend aligns with known microbial growth kinetics, whereby extended assay runtime facilitates microbial detection. In contrast, we observed only a marginal increase in specificity, rising from 57% to 60%, over the same period (Figure 3a). This limited improvement is ascribed to light scattering from the abundant nonbacterial urinary components, such as leukocytes, proteins, and cellular debris, present in the nonprocessed urine. It is worth mentioning that in most rapid UTI detection schemes, the samples are preprocessed by either filtration or centrifugation.^26,32,34–36^ Interestingly, most culture-negative samples (green circles in Figure 3a) exhibited negligible further increases in −Δ*I* (%) beyond 30 min and true bacterial infection signals continued to diverge (Figure 3a). Between 30 and 90 min, 92% of culture-positive samples showed a continuous increase in −Δ*I* (%) and 62% of culture-negative samples exhibited a continued decrease. Despite this trend, the fixed threshold was still insufficient to confidently exclude non-UTI cases.

**Figure 3:**
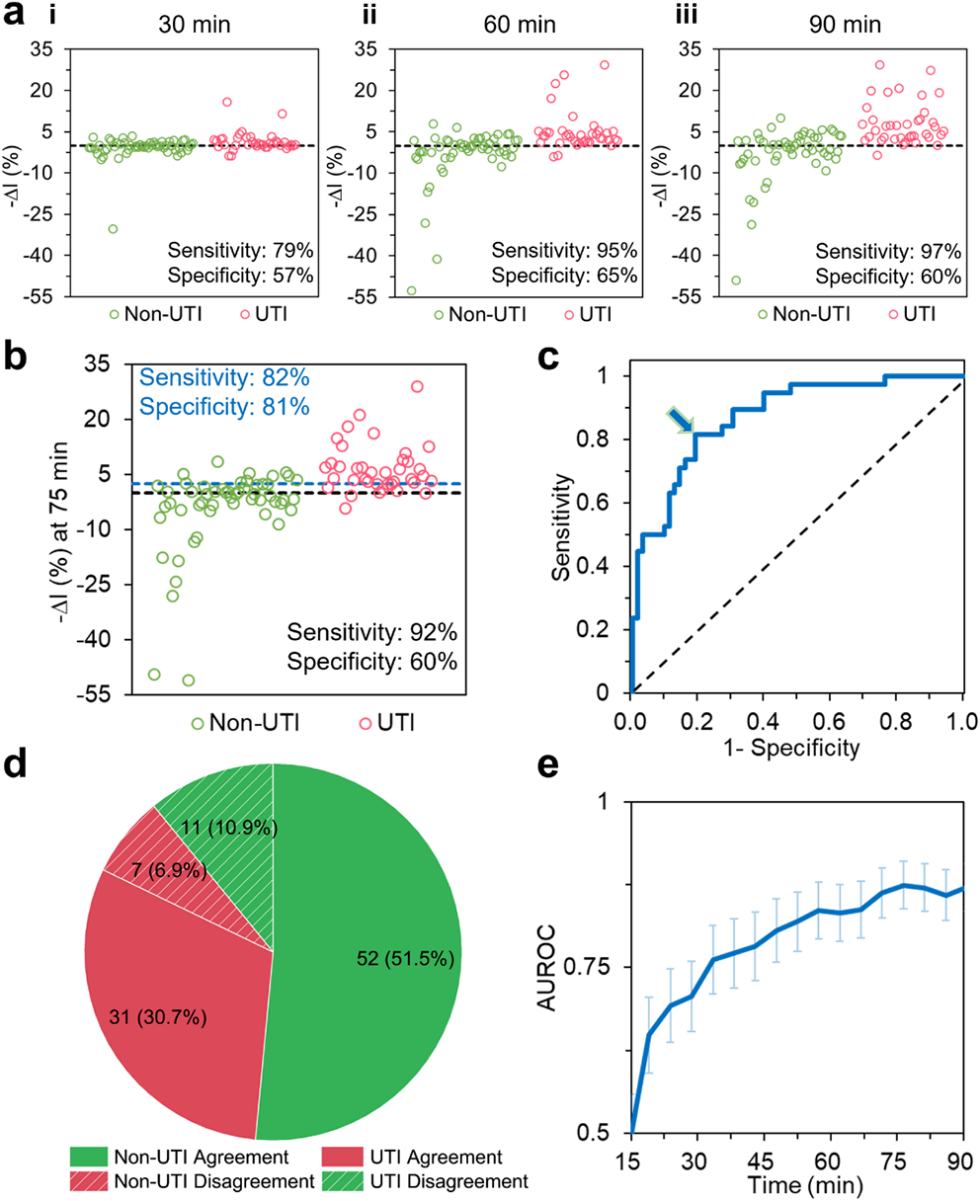
iPRISM for infection screening on fresh untreated clinical urine samples (63 clean samples and 38 bacterial infected samples including the bacterial-fungal coinfection one). (a) Intensity value changes after incubation in iPRISM device for 30, 60, and 90 min. The dashed line represents a double-blind screening threshold of −Δ*I* (%) = 0. Samples surpassing this threshold were considered positive for UTI infection by the iPRISM assay. (b) Intensity value changes at 75 min. The black dashed line represents the original detection threshold of −Δ*I* (%) = 0, while the blue dashed line is the new optimized threshold of −Δ*I* (%) = 2.59. Each circle corresponds to one clinical specimen. Green circles represent specimens identified as noninfected by clinical culture, and red circles represent specimens with positive culture results. (c) ROC analysis at 75 min. The point in the graph indicated by an arrow corresponds to the optimal diagnostic threshold yielding the maximum Youden’s index. (d) Classification agreement at 75 min using the established optimal threshold for data shown in (b). (e) Time-dependent AUC performance. Error bars represent standard error of area under the ROC curve statistics.

To address this issue, we next explored whether optimizing the threshold −Δ*I* (%) value could improve the assay’s specificity by accounting for matrix-induced signal increase, such as those caused by the settling of urine crystals. Receiver Operating Characteristic (ROC) analysis identified an optimal −Δ*I* (%) threshold of 2.59, corresponding to the maximum Youden index, with an area under the curve (AUC) of 0.87 (95% CI: 0.81–0.94; *p* < 0.0001 vs random classification, Figure 3c), which is considered as a strong discriminatory capacity for rapid UTI diagnosis.^68^ At this threshold, diagnostic accuracy reached 83% at 75 min, with balanced sensitivity (82%) and specificity (81%) (Figure 3d). Temporal analysis revealed that the optimal diagnostic performance was at 75 min in the current study (Figure 3e). By comparison, application of the fixed threshold of −Δ*I* (%) = 0 at 75 min yielded moderate overall accuracy of 72%, with 92% sensitivity and 60% specificity (Figure 3b). Together, these results demonstrate that threshold optimization substantially improves the iPRISM assay performance. Although effective, ROC analysis is inherently data-dependent; integrating a machine learning feedback loop could enable dynamic, real-time thresholding to further refine diagnostic accuracy.

Although the primary focus of this study was bacterial detection, an exploratory analysis was conducted to assess iPRISM’s capability in detecting fungal infections. Inclusion of yeast-positive urine in ROC analysis yielded a lower AUC of 0.79 at 75 min (Figure S3), this decline likely stems from multiple factors including the lower clinical thresholds for fungal detection (10–10^3^ CFU mL^–1^ vs 10^5^ CFU mL^–1^ for bacteria), the size mismatch between fungi (1–8 μm) and the chip design, consisting of 3-μm-wide microwells, and the morphology-switching behavior of *Candida albicans*.^69–71^ Additionally, the use of BHI medium, which is optimized for bacterial growth, may hinder fungal proliferation.^72^ Nevertheless, iPRISM correctly distinguished bacterial from fungal infections in 83% of the infected samples at 80 min, excluding a single coinfection case (Figure S3). This result suggests that iPRISM retains discriminatory capability even under nonoptimized conditions for fungal detection, highlighting the inherent flexibility of the platform. Although this segment of the study was not conducted under double-blind conditions and requires prior knowledge of infection type, the current results are promising and build upon our earlier application of iPRISM to antifungal susceptibility testing performed under different experimental settings.^53^ While ongoing efforts aim to extend the platform’s applicability also to fungal detection, such developments are beyond the scope of the current study. Nonetheless, these preliminary observations point toward a feasible pathway for expanding iPRISM to support both bacterial and fungal infection detection, with potential implications for informing timely and targeted therapeutic decisions.^53,72^

The remaining 33 cases involving mixed flora, see Figure 1c, presented substantial diagnostic challenges for iPRISM, reflecting a well-recognized clinical limitation that can complicate diagnosis and delay targeted therapy. In some of these samples, signal increases were observed despite the absence of dominant uropathogens, underlining the inherent ambiguity of mixed microbial populations. In clinical practice (Figure 1b), such ambiguous findings are often resolved through repeated testing. Such mixed flora scenarios also present a diagnostic challenge in clinical setting and delaying the targeted therapy.^73,74^ However, follow-up sample collection was not feasible due to the double-blind study design and delays in receiving reference lab results in the current study. To rule out UTI with sufficient certainty and improve iPRISM assay specificity in infection screening, one promising direction is the application of spatially controlled surface chemical functionalization to the sensing platform^75^ while preserving the capture probe-free nature of the iPRISM platform.^76^

### 3.4 AST Directly from Urine Specimens

iPRISM AST was performed on the same clinical urine specimens in parallel with the UTI screening described above, enabling simultaneous infection detection and assessment of susceptibility to gentamicin and ciprofloxacin within the same iPRISM device as depicted in Figure 1. The assay utilized predetermined MIC breakpoint concentrations of 8 μg mL^–1^ for gentamicin and 0.06 μg mL^–1^ for ciprofloxacin, as established previously,^50^ and values are in line with CLSI (Clinical & Laboratory Standards Institute) guidelines. Results were then compared to a standard AST test performed using the VITEK 2 system, which incurred a minimum two-day delay from specimen submission to reported results.

Figures 4a-i and 4b-i show iPRISM characteristic results for *E. coli* strains isolated from culture positive urine samples. The −Δ*I* (%) values as a function of time are shown for *E. coli* strains that are either resistant or susceptible to gentamicin, measured in the presence or absence of the antibiotic. For the resistant strain (Figure 4a), exposure to gentamicin for 90 min resulted in −Δ*I* (%) values that are comparable to that of the untreated sample. On the other hand, the corresponding −Δ*I* (%) values for the susceptible strain (Figure 4b) are 30% of that of the untreated urine, suggesting growth inhibition. Indeed, HR-SEM images of the retrieved iPRISM chips present reduced numbers of *E. coli* cells for the susceptible strain (Figure 4b-iii) and a dense colonization for the resistant one (Figure 4a-iii), confirming that the optical response reflects phenotypic antibiotic effects.

**Figure 4:**
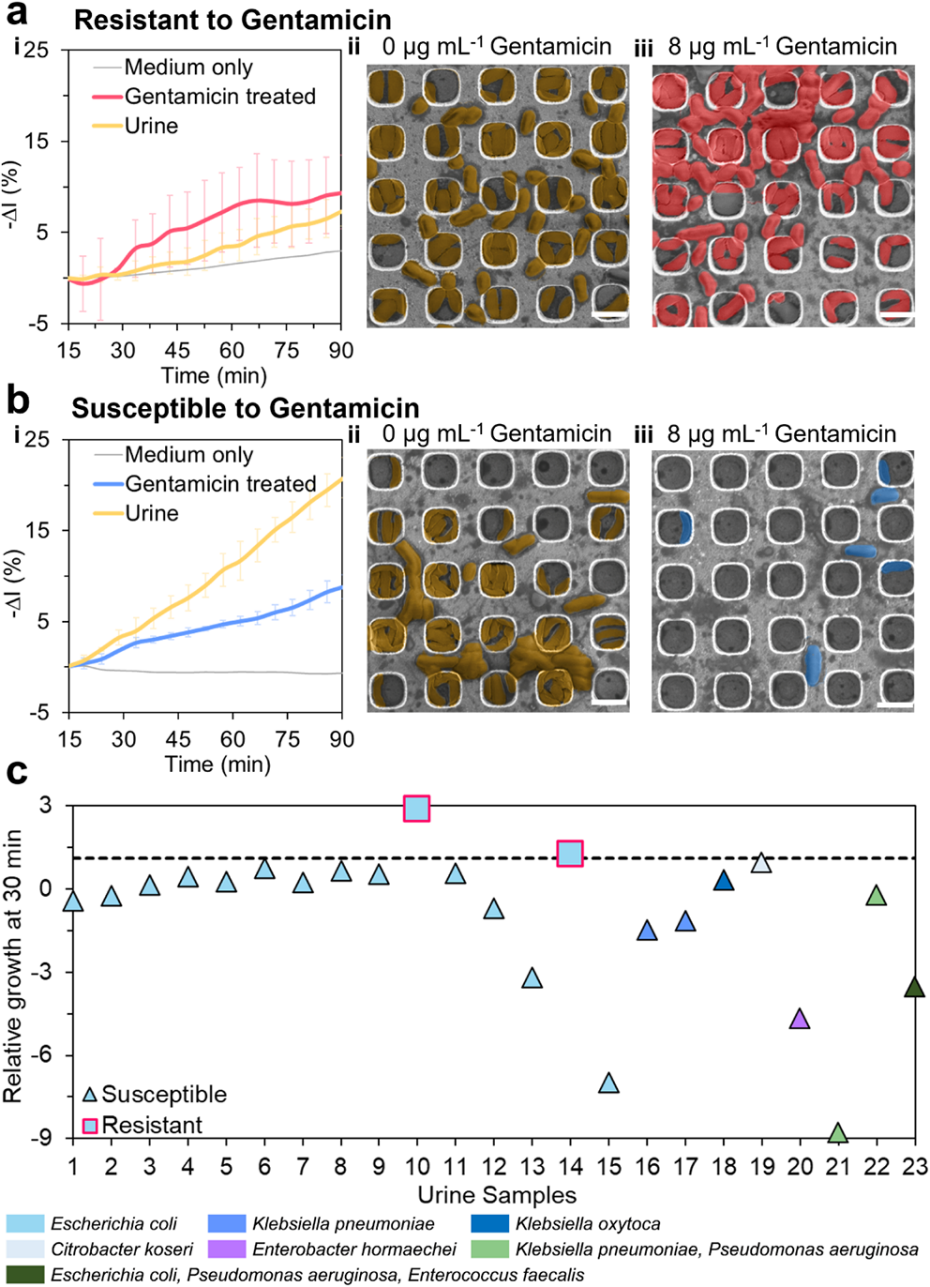
Direct iPRISM AST assay performance in clinical human urine samples with exposure to MIC breakpoint concentration of gentamicin (8 μg mL^–1^). (a, b) Representative gentamicin-resistant (a) and susceptible (b) cases where *E. coli* was isolated from culture positive urine samples. (i) Real-time iPRISM characteristic curves showing −Δ*I* (*%*) values (*n* = 3). (ii, iii) HR-SEM images of (ii) control and (iii) post-treatment. Scale bars represent 3 μm. (c) iPRISM relative growth (RG) values at 30 min after exposure to a gentamicin breakpoint concentration of 8 μg mL^–1^ of urine samples from suspected infected UTI patients (*n* = 23; 2 resistant, 21 susceptible). The dashed line indicates the predefined threshold (RG at 30 min *=* 0.95) for resistance classification.

To quantitatively assess antibiotic resistance in a consistent manner, we calculated the relative growth (RG), defined as the ratio of −Δ*I* (%) in samples exposed to antibiotics and samples without antibiotics at a given time point. Using a previously validated RG threshold of 0.95 for *E. coli*,^50^ iPRISM achieved 100% agreement with the VITEK 2 tests for gentamicin within 30 min across 23 clinical samples (Figure 4c). Specifically, 91.3% of isolates were accurately identified as susceptible, while 8.7% were resistant. Comparable performance was also observed for non-*E. coli* uropathogens, including *Klebsiella* spp. and *Enterobacter hormaechei*. Given that only two resistant isolates were included, further validation in a larger, resistance-balanced cohort is required to confirm assay robustness.

In contrast, ciprofloxacin testing exhibited reduced diagnostic accuracy under the current assay conditions. Among 27 urine specimens evaluated by VITEK 2, 13 were resistant (46.5%) and 15 susceptible (53.5%). At 90 min, iPRISM misclassified 5 resistant samples as susceptible corresponding to a high very major error (VME) rate (38%) and suboptimal sensitivity (62%). Additionally, 2 susceptible isolates were also classified as resistant, resulting in a major error (ME) rate of 13% and specificity of 87% at the pre-established RG threshold. ROC analysis indicated that assay performance improved over time, reaching a maximum AUC of 0.79 at 90 min. However, threshold optimization based on the maximal Youden Index only led to a slight increase in specificity but reduced overall diagnostic performance. Under this adjusted threshold of RG = 0.80, 4 resistant strains were misclassified as susceptible (VME 31%, sensitivity 69%) and 5 susceptible strains as resistant (ME 33%, specificity 67%). Taken together, these results suggest that ciprofloxacin AST performance is constrained by the use of highlight key limitations of using a single ciprofloxacin concentration for AST across multiple species, and by the variability in the initial bacterial load among clinical samples, which are not fully mitigated by threshold optimization alone.

Discrepancies between iPRISM and VITEK 22 results were observed in both Gram-negative (*E. coli*, *Klebsiella pneumoniae*, *Pseudomonas aeruginosa*) and Gram-positive (*Staphylococcus lugdunensis*) species (Figure S4c). In particular, iPRISM did not fully resolve a polymicrobial infection in Sample #27 (Figure S4c), which contained both a ciprofloxacin-resistant *E. coli* and a ciprofloxacin-susceptible *Pseudomonas aeruginosa*, iPRISM classified the sample as resistant. Although this correctly indicated the antibiotic’s ineffectiveness in treating the resistant microorganism, it did not reflect the complex microbial composition.

Ciprofloxacin’s mode of action may further complicate signal interpretation. As a DNA gyrase inhibitor, ciprofloxacin induces DNA damage and elicits a stress response that can transiently suppress growth.^77,78^ This growth arrest can mimic susceptibility for resistant strains in our short-term assays, leading to false-susceptible results. This effect is evident in our analysis of pure *E. coli* infected samples (Figure S4d), where iPRISM showed reduced sensitivity (56%) and a VME rate of 44%. ROC analysis indicated improved performance at an earlier point (45 min) using a substantially lower RG threshold of 0.16, achieving 85% sensitivity and 67% specificity, with only one VME and one ME. These findings suggest that ciprofloxacin-exposed bacteria may undergo delayed growth recovery, complicating rapid AST interpretation.

Moreover, the assay’s reliance on a single ciprofloxacin concentration, which was established for *E. coli*, appears to oversimplify the applicability of iPRISM across diverse bacterial species. For example, in Sample #23, iPRISM classified *Staphylococcus lugdunensis* as resistant, whereas VITEK2 reported susceptibility (MIC ≤ 0.5 μg mL^–1^). Indeed, the ciprofloxacin concentration tested in iPRISM was 0.06 μg mL^–1^ which falls below the CLSI-defined susceptibility breakpoint range for staphylococci (0.12–0.5 μg mL^–1^).^79^ At this subinhibitory concentration, even susceptible strains may not exhibit sufficient phenotypic inhibition within the assay’s time frame, leading to apparent resistance and misclassification. In contrast, the gentamicin concentration employed in iPRISM aligned well with CLSI breakpoints for Enterobacteriaceae,^79^ likely contributing to the assay’s consistent classification accuracy. These observations underscore the need to tailor antibiotic concentrations and MIC calculation to a broader range of pathogens for improving diagnostic precision, particularly in polymicrobial infections.

Importantly, the study highlights a key operational insight, while iPRISM integrates simultaneous infection detection and AST on a single optical platform, each function operates optimally at different readout times. Infection screening is more effective after extended incubation (75–90 min), allowing for more distinct microbial colonization signals. Whereas, AST performance for gentamicin is optimal at much earlier time points (within 30 min), driven by rapid phenotypic responses to antibiotic exposure. This difference reflects a flexible and adaptive feature of the platform, enabling a significant time saving and streamlined workflows compared to the current stepwise and lengthy clinical workflow (Figure 1b).

Overall, iPRISM demonstrates significant promise for rapid, phenotypic AST directly from clinical urine samples, particularly for gentamicin. However, performance for ciprofloxacin was less robust under the current assay conditions. This outcome is ascribed to the iPRISM’s directfrom-sample workflow, which omits several conventional AST steps such as species identification,^80^ inoculum standardization,^80–82^ MIC determination,^80,83^ and breakpoint-based classification.^29,80,83,84^ While our streamlined approach offers rapid testing and faster results, it should be refined to mitigate these challenges.

Future efforts should focus on expanding clinical validation with a larger, resistancebalanced cohort and integrating automated microfluidics to enable multiplexed AST profiling using multiple antibiotics across varied concentraions, and incorporate species identification for obtaining correct species-specific AST dignostic results.

## 4 Conclusions

To the best of our knowledge, this study represents the first direct-in-urine double-blind clinical study to integrate infection detection and AST in a single real-time assay, omitting the need of prior preprocessing. This integration directly addresses a major bottleneck in current UTI diagnostics by coupling pathogen detection and therapeutic guidance within a single step. This prospective clinical study conducted at the near-patient bedside and enrolled a complex inpatient population including cases with preantibiotic exposure, polymicrobial growth and complicated UTIs, which sets a new benchmark for POC integrated UTI diagnostics.

The iPRISM platform achieves rapid, culture-independent integrated UTI screening and AST within a streamlined assay. It achieved 97% sensitivity in bacteriuria screening within 90 min and 100% sensitivity and specificity for gentamicin susceptibility profiling within just 30 min. These performance metrics demonstrate that clinically actionable results can be obtained on time scales relevant to initial treatment decision-making.

Notably, preliminary data indicate that iPRISM can distinguish bacterial from fungal infections, which is an important yet currently unmet diagnostic need, given their distinct treatment strategies required and the growing recognition of invasive fungal infections as a public health threat.^8,85–87^

Furthermore, current validation focused on inpatient populations including a substantial proportion of complicated UTIs and polymicrobial infections. We anticipate superior performance in outpatient care settings, where uncomplicated and *E. coli*-associated UTIs are more prevalent.^60,61^ To enhance the reliability and generalizability of diagnostic findings, future multicenter studies should include outpatient and community-based settings, a balanced distribution of resistance phenotypes, and additional reference standards. Such studies will be essential for defining clinical utility across diverse care environments.

Building on these strengths, future development will be directed toward four critical areas: (1) mitigating matrix-induced signal artifacts and resolving mixed flora interference through chemo-micropatterned silicon arrays, dynamic growth profiling, and adaptive machine learning; (2) enabling high-throughput MIC determination and expand AST capabilities via an automated, multiplexed platform that generates dynamic concentration gradients; (3) rationally optimizing experimental conditions for broader applicability across diverse bacterial and fungal pathogens; and (4) further miniaturization and integration of the platform that could support true POC deployment. Together, these efforts aim to improve robustness, scalability, and translational readiness of the technology.

By delivering actionable results within clinically relevant timeframes during the first visit without displacing existing clinical workflows, iPRISM offers a practical and impactful contribution to precision infectious disease management and global antimicrobial stewardship.^8,87^

## Supplementary Material



## Notes

Study participant demographics; iPRISM principles; double-blinded urine test results for microbial infections; differentiation between bacterial and fungal infections using iPRISM; iPRISM-based AST for ciprofloxacin in bacterially infected urine samples (PDF)
