# Supplementary material for: Integrated iPRISM Direct-on-Urine Platform for Rapid UTI Diagnosis in a Double-Blind Clinical Trial
Source: ACS Meas Sci Au. 2026 Feb 18;6(2):454–65. doi: 10.1021/acsmeasuresciau.5c00187 (PMC13087939; doi:10.1021/acsmeasuresciau.5c00187)
Supplement: Supplementary file 1 [file tg5c00187_si_001.pdf]

Supporting Information

Integrated iPRISM Direct-on-Urine Platform for Rapid UTI Diagnosis in a Double-Blind Clinical Trial

*Xin Jiang[a], Ramy Fishler[a], Gali Ron[a], Keren Boguslavsky[b], Sarel Halachmi[b,c], and Ester Segal[a]**

[a] *Department of Biotechnology and Food Engineering, Technion – Israel Institute of Technology, Haifa 3200003, Israel*

[b] *Department of Urology, Bnai Zion Medical Center, Haifa 3104800, Israel*

[c] *The Faculty of Medicine, Technion – Israel Institute of Technology, Haifa 3525433, Israel*

*Corresponding author: esegal@technion.ac.il

Table S1: *Study participants demographics of 144 patients.*

	Number of Samples
**Age(years)**	
**0-39**	17 (12 %)
**40-79**	100 (69 %)
**>80**	27 (19 %)
**Gender**	
**Male**	91 (63 %)
**Female**	53 (37 %)

Note: Two samples were from one female of age 46 (spontaneous urination and nephrostomy

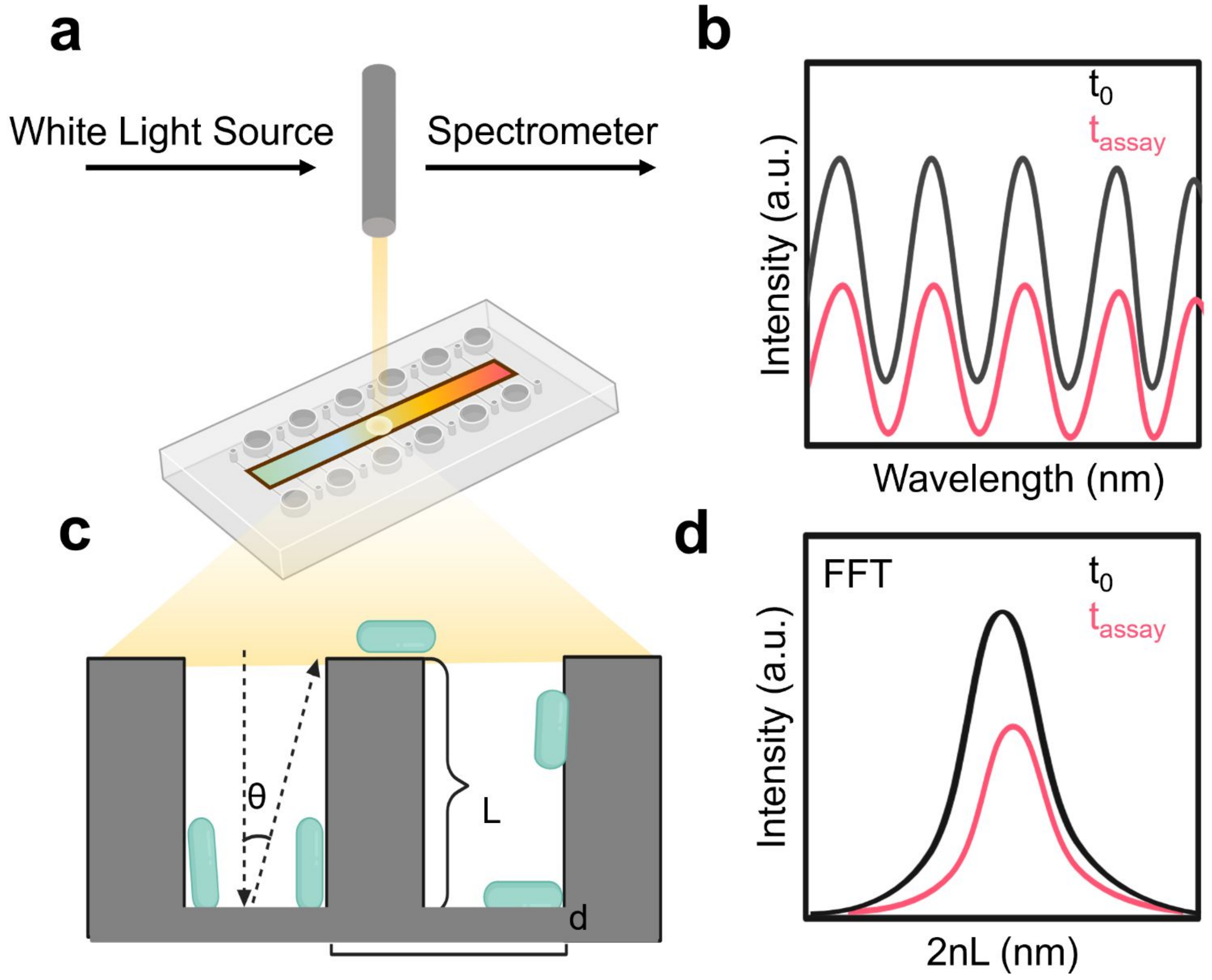

Figure S1: *Principle of iPRISM based on photonic silicon chips consisting of microwell diffraction gratings. (a) Photonic silicon chips integrated in a custom microfluidic cassette are illuminated by a white light source position at a normal angle, and reflected spectra are collected via a CCD spectrometer. (b) The resulting reflectance spectra exhibit characteristic interference fringes reflected from the top and bottom of the microwells. (c) Schematic illustration of the reflectance from silicon microstructure. Photonic silicon chips act as a binary grating in the zero-order (θ=0) of the diffraction pattern. L represents the height of the microstructure, and d is its width. (d) Reflectance spectra are analyzed via Fast Fourier Transform (FFT), generating a characteristic peak, in which the peak amplitude corresponds to the intensity of the reflected light and the peak position to the 2nL value and sensitive to where microbial adhere to the porous silicon surface. Created with BioRender.*

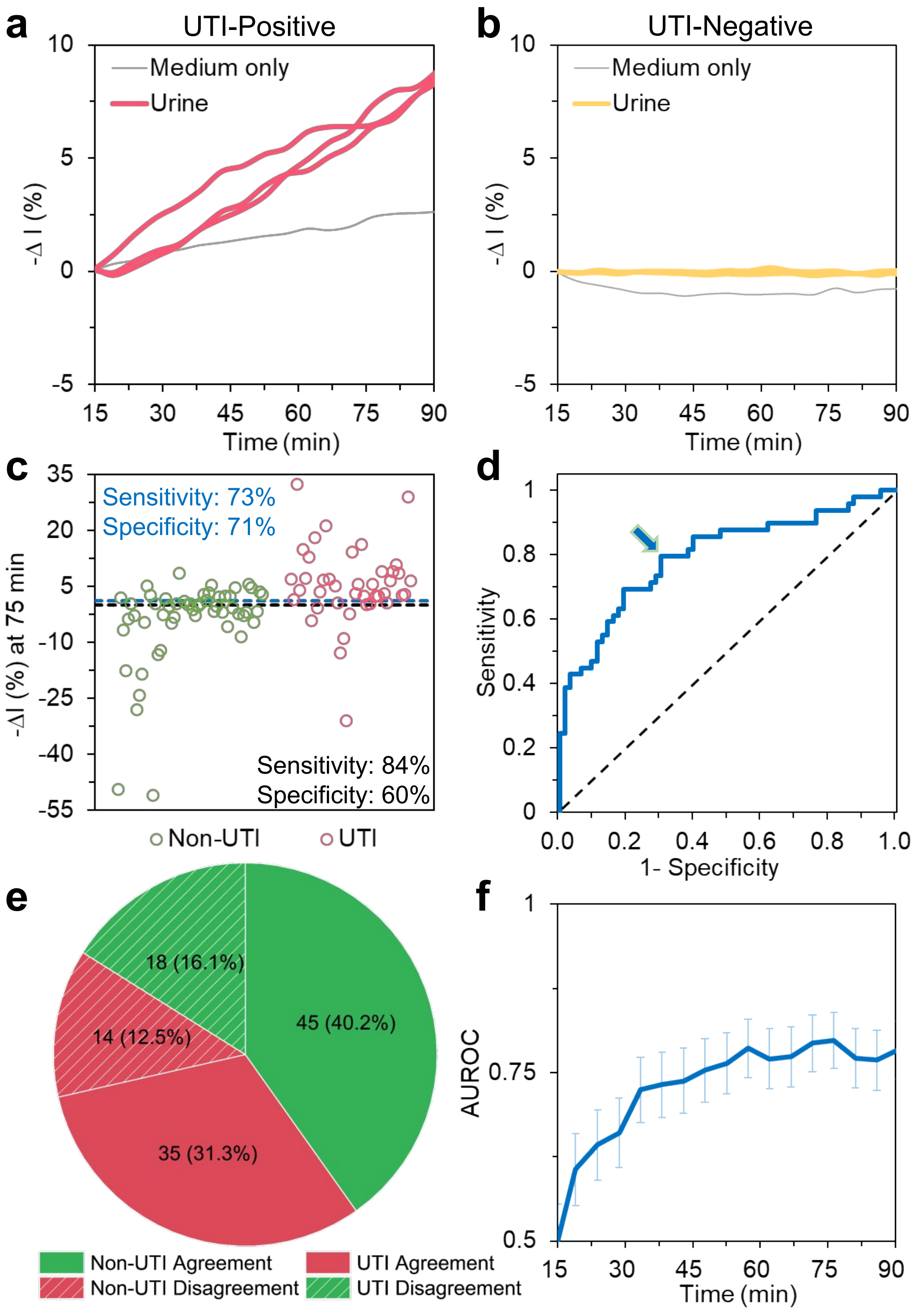

Figure S2: *iPRISM for double-blind infection screening directly on clinical urine samples (63 clean samples and 49 microbial infected samples). (a,b) Representative characteristic iPRISM growth curves of (a) UTI-positive and (b) UTI-negative samples (n=3). (c) Intensity changes at 75 min (thresholds: black (threshold set at -ΔI (%) = 0), blue (optimal threshold found via ROC analysis in d)). (d) ROC analysis at 75 min for determining the optimal diagnostic threshold - Youden's index (arrow). (e) Classification agreement using the established threshold. (f) Time-dependent AUC performance with standard error, plateauing at 0.79 after 75 min.*

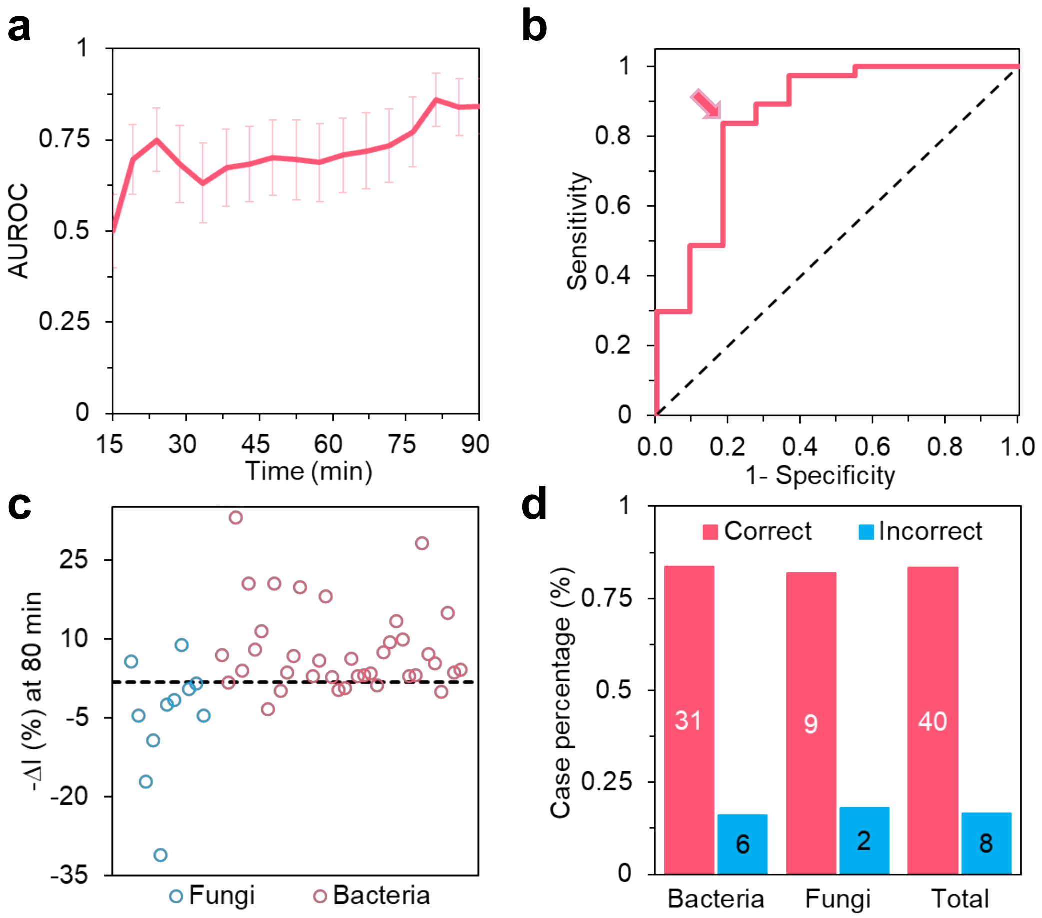

Figure S3: *iPRISM for bacterial and fungal infection screening directly on clinical urine samples (37 bacterial infected samples and 11 fungal infected samples). (a) Time-dependent AUC performance with standard error. Performance plateaus at an AUC of 0.86 after 80 min. (b) ROC curve at 80 min for determining Youden's index (optimal diagnostic threshold, arrow). (c) Intensity value changes at 80min. The dashed line indicates the cutoff derived from the ROC curve in (b). (d) Classification agreement at the established diagnostic threshold with the number of cases marked in the center of the bar at 80 min. Note that the bacteria-fungal co-infection sample was excluded from this study analysis.*

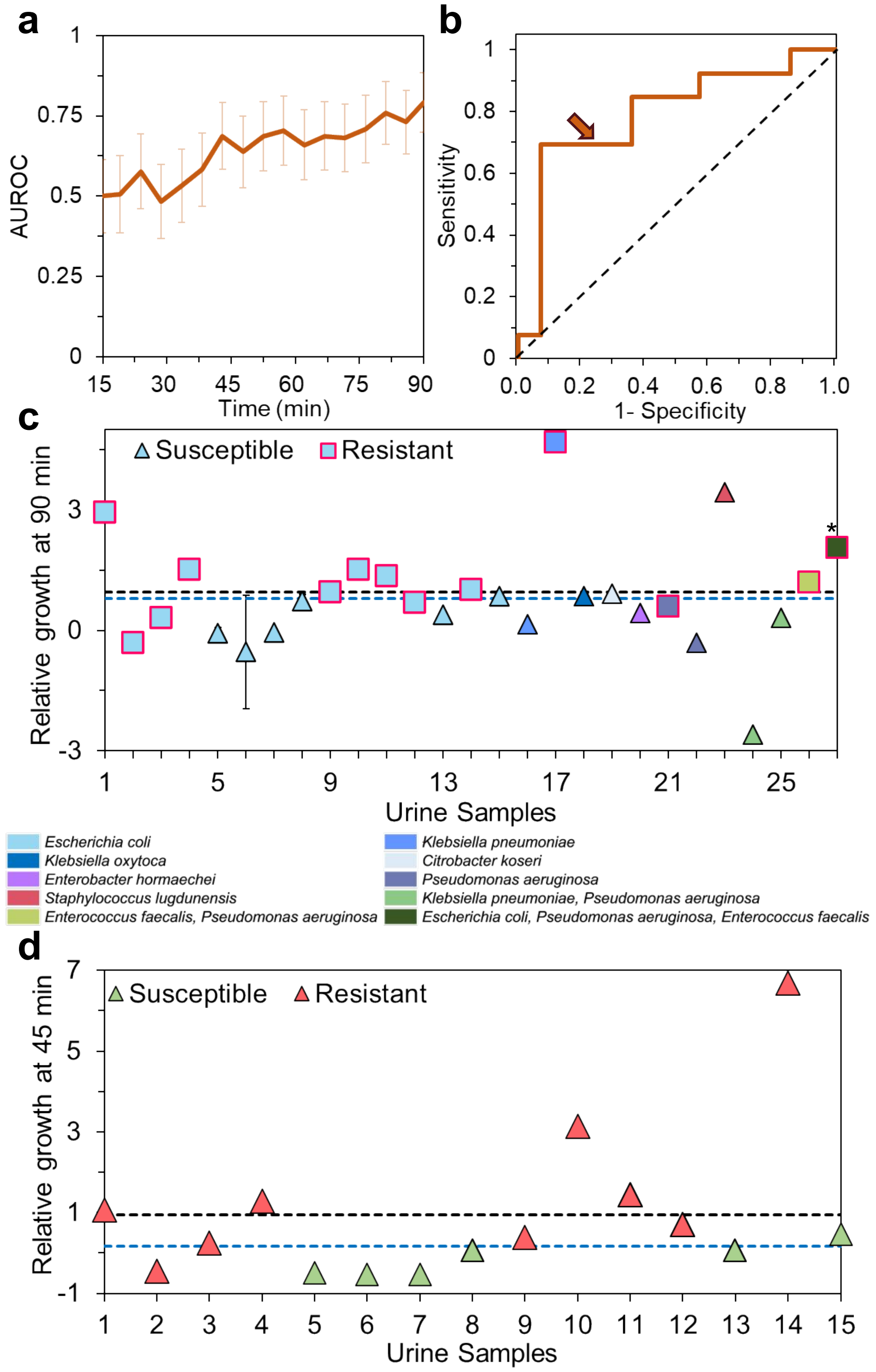

Figure S4: *Direct iPRISM AST assay performance in clinical human urine samples with exposure to MIC breakpoint concentration of ciprofloxacin (0.06 μg mL^{-1}). (a) Time-dependent AUC performance with standard error. (b) ROC curve at 90 min for determining optimal diagnostic threshold for susceptibility classification. (c) iPRISM relative growth (RG) values at 90 min after exposure to a ciprofloxacin breakpoint concentration of 0.06 μg mL^{-1} of urine samples from suspected infected UTI patients. (n = 28; 14 resistant, 14 susceptible). Dashed lines indicate the predefined threshold (RG = 0.95, black) and ROC-optimized threshold (RG = 0.80, blue). * for the sample with both resistant E. coli infection and susceptible P. aeruginosa infection. (d) iPRISM relative growth (RG) values at 45 min after exposure to a ciprofloxacin breakpoint concentration of 0.06 μg mL^{-1} of urine samples from suspected infected UTI patients. (n = 15; 9 resistant, 6 susceptible). Dashed lines indicate the predefined threshold (RG = 0.95, black) and ROC-optimized threshold (RG = 0.16, blue).*
